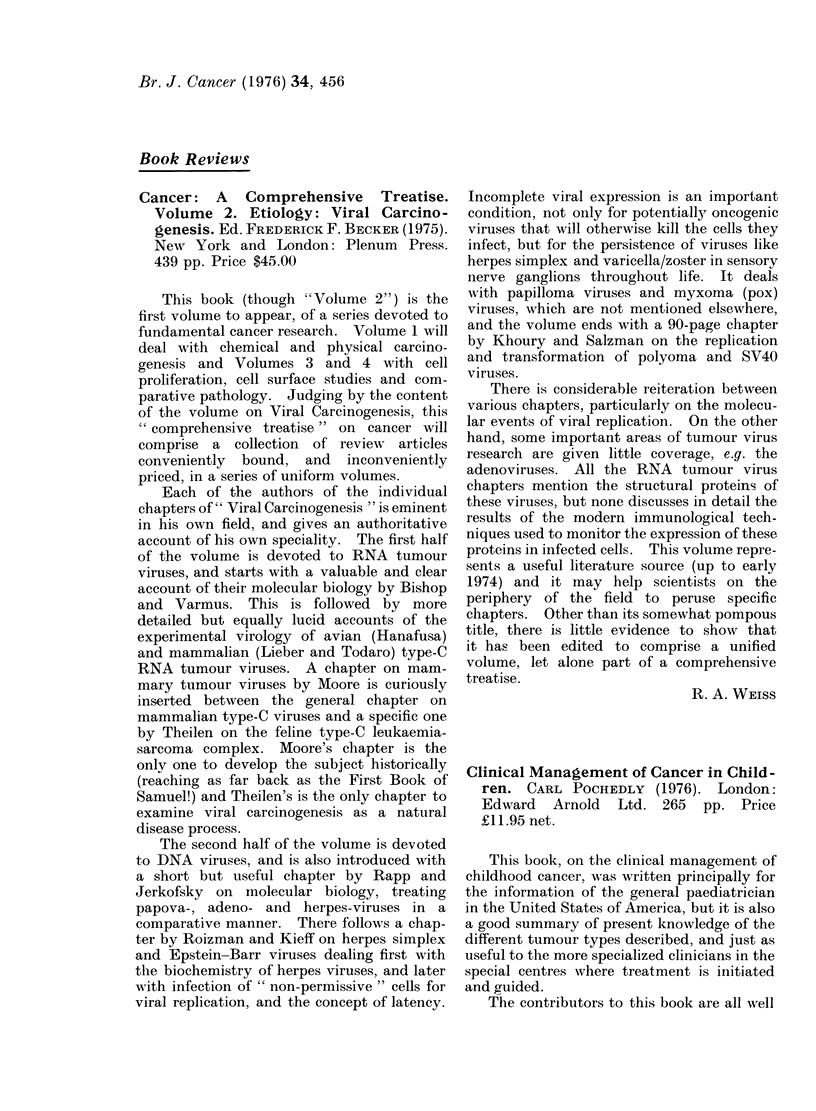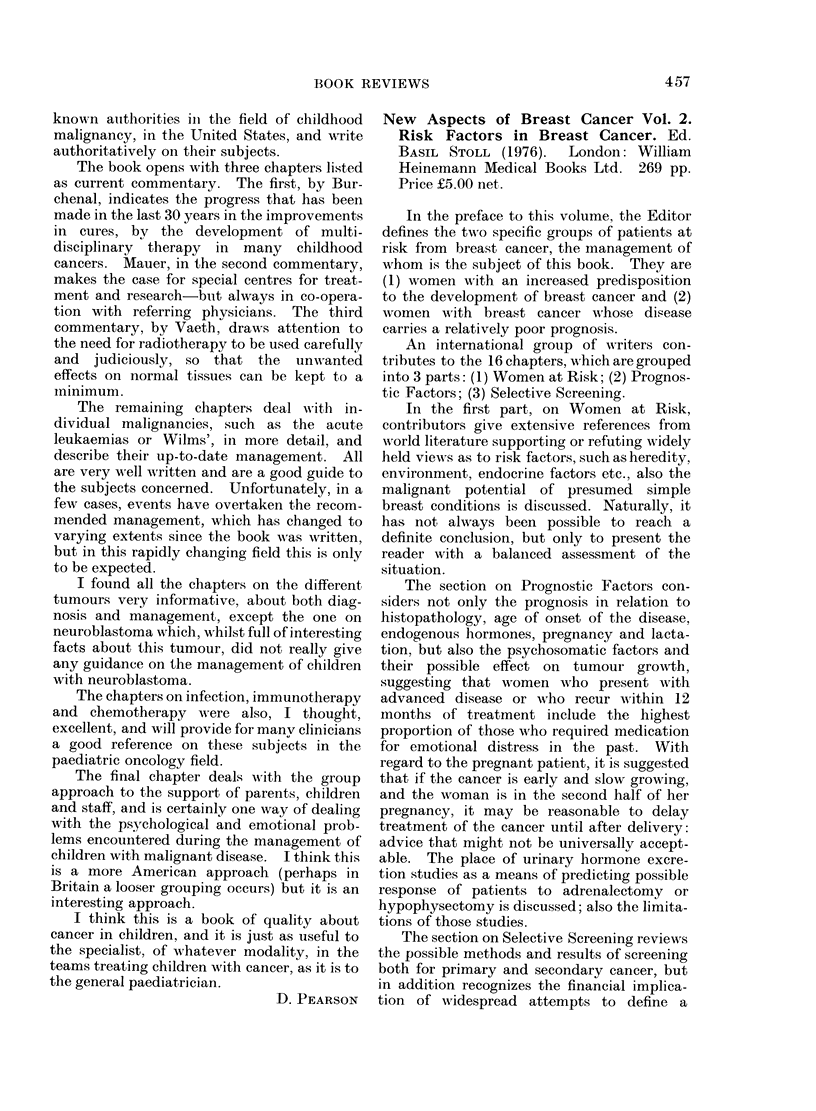# Clinical Management of Cancer in Children

**Published:** 1976-10

**Authors:** D. Pearson


**Clinical Management of Cancer in Children.** CARL POCHEDLY (1976). London: Edward Arnold Ltd. 265 pp. Price £11.95 net.

This book, on the clinical management of childhood cancer, was written principally for the information of the general paediatrician in the United States of America, but it is also a good summary of present knowledge of the different tumour types described, and just as useful to the more specialized clinicians in the special centres where treatment is initiated and guided.

The contributors to this book are all well known authorities in the field of childhood malignancy, in the United States, and write authoritatively on their subjects.

The book opens with three chapters listed as current commentary. The first, by Burchenal, indicates the progress that has been made in the last 30 years in the improvements in cures, by the development of multidisciplinary therapy in many childhood cancers. Mauer, in the second commentary, makes the case for special centres for treatment and research—but always in co-operation with referring physicians. The third commentary, by Vaeth, draws attention to the need for radiotherapy to be used carefully and judiciously, so that the unwanted effects on normal tissues can be kept to a minimum.

The remaining chapters deal with individual malignancies, such as the acute leukaemias or Wilms', in more detail, and describe their up-to-date management. All are very well written and are a good guide to the subjects concerned. Unfortunately, in a few cases, events have overtaken the recommended management, which has changed to varying extents since the book was written, but in this rapidly changing field this is only to be expected.

I found all the chapters on the different tumours very informative, about both diagnosis and management, except the one on neuroblastoma which, whilst full of interesting facts about this tumour, did not really give any guidance on the management of children with neuroblastoma.

The chapters on infection, immunotherapy and chemotherapy were also, I thought, excellent, and will provide for many clinicians a good reference on these subjects in the paediatric oncology field.

The final chapter deals with the group approach to the support of parents, children and staff, and is certainly one way of dealing with the psychological and emotional problems encountered during the management of children with malignant disease. I think this is a more American approach (perhaps in Britain a looser grouping occurs) but it is an interesting approach.

I think this is a book of quality about cancer in children, and it is just as useful to the specialist, of whatever modality, in the teams treating children with cancer, as it is to the general paediatrician.

D. Pearson